# Risk of harm from alcohol use and heavy alcohol consumption: Its association with other NCD risk factors in Thailand

**DOI:** 10.1038/s41598-019-52754-w

**Published:** 2019-11-08

**Authors:** Nalinee Jakkaew, Kanokporn Pinyopornpanish, Wichuda Jiraporncharoen, Anawat Wisetborisut, Surin Jiraniramai, Ahmar Hashmi, Chaisiri Angkurawaranon

**Affiliations:** Department of Family Medicine, Faculty of Medicine, Chiang Mai University, Chiang Mai, Thailand, 110 Intawaroros Road, Muang, Chiang Mai 50200 Thailand

**Keywords:** Cardiovascular diseases, Risk factors

## Abstract

While there is an abundance of literature examining the relation between quantity of alcohol consumption and risk factors for non-communicable diseases (NCD), there is less evidence on whether the risk of harm from alcohol use would have a similar relationship with NCD risk factors. The study aims to determine the association between level of harm from alcohol use and NCD risk factors. A cross-sectional survey was conducted among health care workers in Thailand in 2013. The Alcohol, Smoking and Substance Involvement Screening Test (ASSIST) was used to assessed risk of harm from alcohol use. The results suggest that higher risk of harm from alcohol use was associated with two of the eight NCD risk factors among women (higher blood pressure and higher triglyceride level) and five of the eight NCD risk factors among men (smoking, physical inactivity, higher blood pressure, higher blood glucose and higher triglyceride level). For men, assessing risk of harm could be incorporated as part of NCD programs as practitioners do not have to worry about the accuracy of the alcohol quantification and conversion to standard drinks. However, among women, quantifying volume may still be needed.

## Introduction

Non-communicable diseases (NCD) are a current global health concern with an estimated thirty-eight million people dying from NCD each year. Fifteen million NCD deaths are premature with more than 80 percent occurring in low and middle income countries (LMIC), which includes Thailand^1,2^. According to the World Health Organization (WHO), harmful use of alcohol is considered one of the key shared risk factors for the four major NCD: cardiovascular disease, cancers, chronic respiratory diseases and diabetes^1,3^, for which risk factors such as alcohol drinking, male sex, age, high blood pressure and body mass index (BMI) play a synergistic role in disease incidence^4,5^.

Aspects of alcohol use that play a role in harmful outcomes relate to the volume, pattern^6^, and concentration of alcohol within drinks; drinking context; and behaviors influenced such as driving when drunk. Currently, there are many screening tools to assess alcohol use. The Alcohol Use Disorder Identification Test (AUDIT)^7^ is one of the screening tools suggested by WHO that measures volume and pattern of consumption through standardized alcoholic concentrations of consumed alcoholic beverages, known as the ‘standard drink’. Another WHO instrument to assess alcohol use in relation to other NCD risk factors is the WHO STEPS instrument^8^. The STEPS instrument assesses frequency and pattern of alcohol use as with the AUDIT screen, however, the use of both screening tools are limited in practice, quantifying alcohol use requires conversion of different types of alcohol and alcoholic beverages to the standardized alcohol content recommended by these tools.

In Thailand and other developing countries, another screening tool, the Alcohol, Smoking and Substance Involvement Screening Test (ASSIST)^9^ is also recommended by the WHO for screening non-prescription drug use such as alcohol in primary care. ASSIST is short and not complicated to use as it does not specify the volume or pattern but captures the risk of harm from alcohol use through loss of personal and social functions. The risk of harm from alcohol use is categorized into three levels: low risk, moderate risk and high risk. The appropriate brief interventions for each category of harm is also included in the ASSIST manual^10^ which makes it easier to implement in primary care.

While there is an abundance of literature examining the relation between pattern and quantity of alcohol use and NCD diseases and risk factors^6,11,12^, there is less evidence on whether the risk of harm from alcohol use captured through ASSIST would have a similar relationship with NCD risk factors in LMIC such as Thailand. This cross-sectional study conducted in Thailand aims to determine the association between level of harm from alcohol use, as measured using ASSIST and other NCD risk factors. The study also compared the sensitivity of ASSIST to WHO STEPS in determining harmful alcohol use in this population.

## Methods

### Study design

A cross-sectional survey was conducted among health care workers employed by the Faculty of Medicine of Chiang Mai University and Maharaj Nakorn Chiang Mai Hospital between January 2013 and June 2013. A detailed protocol of the survey has been published^13^. In summary, for their annual or periodical check-up, participants answered an online questionnaire regarding their personal details such as age, gender and income. On the day of their physical examination, self-administered ASSIST questionnaire was given to the participants. Participants were then interviewed by trained investigators not employed by Faculty of Medicine CMU or Maharaj Nakhorn Hospital, on their NCD risk factors according to the WHO STEPS instruments. Afterwards, the participants received a physical examination where their anthropometric measurements were taken and venous blood samples were taken for laboratory analyses.

### Measures of alcohol consumption and harm

Risk of harm from alcohol use: The ASSIST was used to categorize the level of harm in the past three months into three categories: (1) Lifetime abstainers, (2) low risk of harm and (3) moderate and high risk of harm. The Thai version of the ASSIST questionnaire has been validated and used in published literature^13^.

Pattern of alcohol use: The WHO STEPS questionnaire was used to categorized pattern of alcohol consumption within the past month into three groups: (1) lifetime abstainer (2) non-binge drinker (<4 standard drinks per occasion for women and <5 standard drinks per occasion for men) and (3) binge drinker (≥4 standard drinks per occasion for women and ≥5 standard drinks per occasion for men).

### Other risk factors for NCDs

The WHO STEPS questionnaire was used to quantify the following behavioral risk factors for NCD consisting of:

Tobacco use: Patients were categorized as either current smoker or non-smoker.

Insufficient physical activity: WHO recommends that to achieve sufficient physical activity for health, any combination of moderate- and/or vigorous- intensity activities should achieve a minimum of at least 600 metabolic equivalent of task (MET)-minutes per week. Those failing to achieve at least 600 MET minutes per week, were considered as having inadequate physical activity.

Insufficient fruit and vegetable consumption: Participants eating less than five servings of fruit and/or vegetables per day is categorized eating insufficient amount of fruit and vegetable consumption.

Other physiological risk factors for NCDs include high body mass index (BMI), high blood pressure (BP), high fasting blood sugar level (FBS) and high cholesterol specifically high low density lipoprotein (LDL) cholesterol and high triglycerides (TG).

### Other factors of interests

From a review of literature, key potential confounding factors between alcohol use and other NCD risk factors were included, such as age, sex, education, income and occupation. All these factors were measured though the self-administered online questionnaire. In addition, biological markers of liver injury and alcohol use, serum alanine aminotransferase (ALT) and aspartate transaminase (AST) were also measured in the physical exam and venous sampling. These two markers usually rise among those with alcoholic hepatitis and heavy alcohol use.

### Ethics and consent

The Ethics approval was obtained from the Faculty of Medicine, Chiang Mai University (CMU) No 069/2012 in accordance with the guideline and regulation of Chiang Mai University. Informed consent was obtained from all participants.

### Statistical analysis

Descriptive statistics were used to describe basic demographic data. Alcohol consumption and patterns of alcohol consumption were compared between men and women using t-test, Wilcoxon rank sum test or chi-square. To explore the associations between harmful alcohol use and other risk factors for NCDs, linear regression was used for continuous outcomes and logistic regression were used for binary outcomes. Age and sex were considered *a priori* confounders and all analyses were stratified by sex and adjusted for age. Additional adjustments for other potential confounders including education, income, and occupation were also done using regression analyses. The p-value from the overall likelihood ratio test for the association between the main exposure (alcohol consumption or harm) and that NCD risk factor were obtained.

## Results

In total, there were 3,204 participants who completed the survey, representing a 60% response rate from the total eligible population. Among the participants, 1,394 (43.5%) were lifetime abstainers from alcohol use. Over ninety percent of abstainers were female. Among drinkers, 80.7% drank alcohol at low risk of harm and 19.3% were moderate to high risk. Three-fourths of low risk drinkers were female. Demographic data categorized by risk of harm from alcohol use were described in Table 1. Drinkers at moderate to high risk of harm from alcohol use tended to be younger with lower education and income (Table 1).

**Table 1:** Risk of Harm from alcohol use.

	Female (2,472)			Male (732)			p-value*
	Abstainer (1,296)	Low risk (1,091)	Moderate/High risk (85)	Abstainer (98)	low risk (370)	Moderate/High risk (264)	
Mean age (sd)	42.6 (10.7)	37.4 (10.5)	34.7 (9.7)	44.6 (10.4)	41.3 (10.0)	38.2 (9.0)	0.32**
Highest education (col %)							<0.001#
Below bachelor’s degree	34.5	21.5	47.0	55.1	48.4	68.2	
Bachelor’s degree	51.1	66.5	51.8	33.7	40.0	29.2	
Higher than bachelor’s degree	14.4	12.0	1.2	11.2	11.6	2.6	
Income per month (col %)							<0.01^#^
<20,000	30.7	30.5	54.1	56.1	47.6	71.2	
20,000–40,000	28.9	33.1	35.3	23.5	24.9	17.4	
40,000–60,000	19.8	18.3	4.7	9.2	10.0	6.1	
>60,000	20.6	18.1	5.9	11.2	17.6	5.3	
Job (col %)							<0.01^#^
Doctors/nurses	46.5	56.4	22.3	19.4	23.2	4.6	
Other health professionals	24.4	19.3	24.7	16.3	17.3	12.1	
Administrators	10.9	9.5	16.5	18.4	13.2	11.4	
Workers	18.2	14.8	36.5	45.9	46.2	72.0	

^*^p-value comparing demographic status between men and women; **T-test; ^#^Chi-square.

### Relationship between risk of harm from alcohol use and heavy alcohol drinking

The average number of standard drinks per day for drinkers at low risk of harm was 1.03 for women and 2.98 drinks for men. The average number of standard drinks per day for those at moderate to high risk of harm was 3.56 for women and 7.05 for men. Almost 10% of women at low risk of harm from alcohol were binge drinkers. About 44% of women at high risk of harm from alcohol use were binge drinkers while over 80% of men at high risk of harm from alcohol use were binge drinkers (Table 2).

**Table 2:** Risk of Harm from alcohol use and patterns of alcohol use.

	Female (2,472)			Male (732)			p-value*
	Risk of Harm from alcohol use			Risk of harm from alcohol use			
	Abstainer (1,296)	Low risk (1,091)	Moderate/High risk (85)	Abstainer (98)	low risk (370)	Moderate/High risk (264)	
Alcohol consumption							
Mean number of standard drink per day in last 30 days (sd)	0 (0)	1.03 (1.93)	3.56 (4.07)	0 (0)	2.98 (3.59)	7.05 (5.5)	<0.01**
Median number of standard drink per day in past 30 days (IQR)	0 (0–0)	0 (0–1)	2.5 (1–5)	0 (0–0)	2 (0–5)	6 (3–10)	<0.01^##^
Pattern of alcohol consumption (col %)							<0.01^#^
Abstainer	100	0	0	100	0	0	
Non-binge drinking	0	90.4	55.3	0	65.1	17.4	
Binge drinking	0	9.6	44.7	0	34.9	82.6	
Biological marker							
Mean AST (sd)	22.5 (8.2)	22.3 (8.2)	21.7 (5.1)	26.1 (6.5)	29.8 (15.8)	39.0 (28.7)	<0.01^**^
Mean ALT (sd)	19.2 (12.9)	18.5 (12.3)	18.3 (8.8)	27.6 (13.3)	34.1 (26.0)	46.1 (41.0)	<0.01^**^

^*^p-value comparing alcohol consumption and patterns of alcohol consumption between men and women; ^**^T-test; ^#^Chi-square; ^##^Wilcoxon rank-sum test.

### Risk of harm from alcohol use and risk factors for NCD

**Figure 1:**
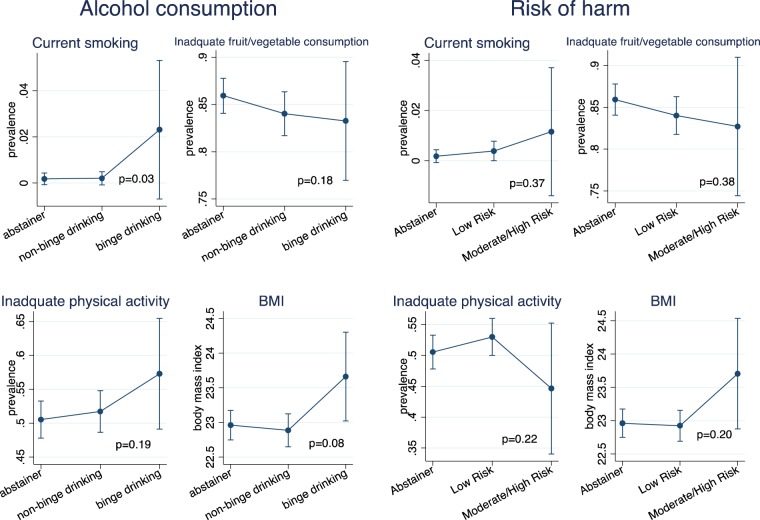
Heavy alcohol use, risk of harm from alcohol use and behavioral risk factors for NCDs among women (N = 2,472). p-value obtained from overall likelihood ratio test between exposure of interests (alcohol consumption or risk of harm) and NCD risk factor using linear or logistic regression. Error bars represent 95% Confidence intervals.

**Figure 2:**
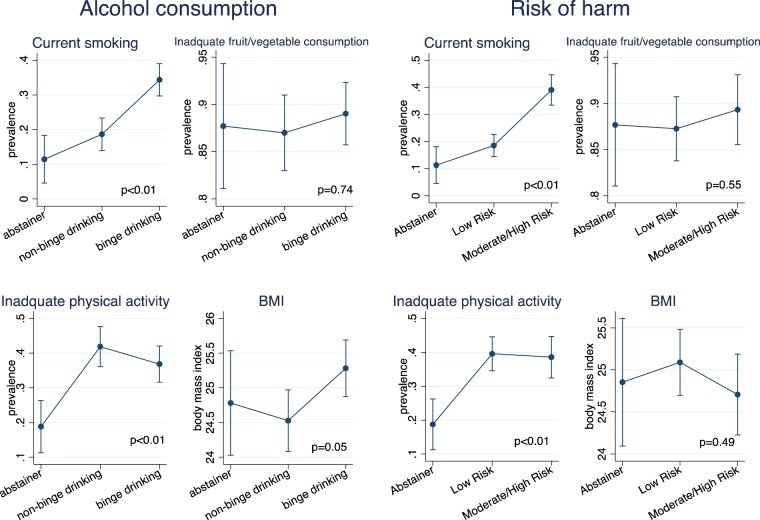
Heavy alcohol use, risk of harm from alcohol use and behavioral risk factors for NCDs among men (N = 732) p-value obtained from overall likelihood ratio test between exposure of interests (alcohol consumption or risk of harm) and NCD risk factor using linear or logistic regression. Error bars represent 95% Confidence intervals.

In the multivariable model adjusting for age, income, education and occupation, higher risk of harm from alcohol use was not significantly associated with current smoking, inadequate fruit and vegetable consumption, physical activity and BMI among women (Fig. 1). For men, higher risk of harm from alcohol use was associated with current smoking and inadequate physical activity but not associated with inadequate fruit/vegetable consumption or BMI (Fig. 2).

**Figure 3:**
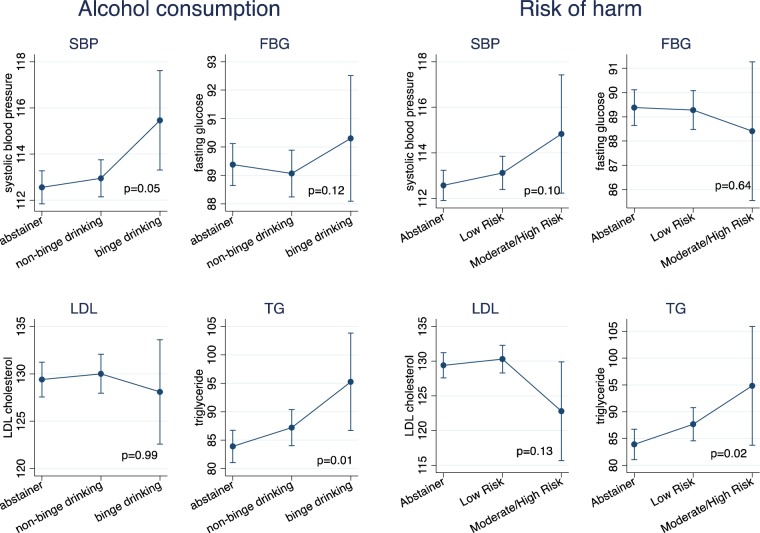
Heavy alcohol use, risk of harm from alcohol use and physiological risk factors for NCDs among women (N = 2,472). p-value obtained from overall likelihood ratio test between exposure of interests (alcohol consumption or risk of harm) and NCD risk factor using linear or logistic regression. Error bars represent 95% Confidence intervals.

**Figure 4:**
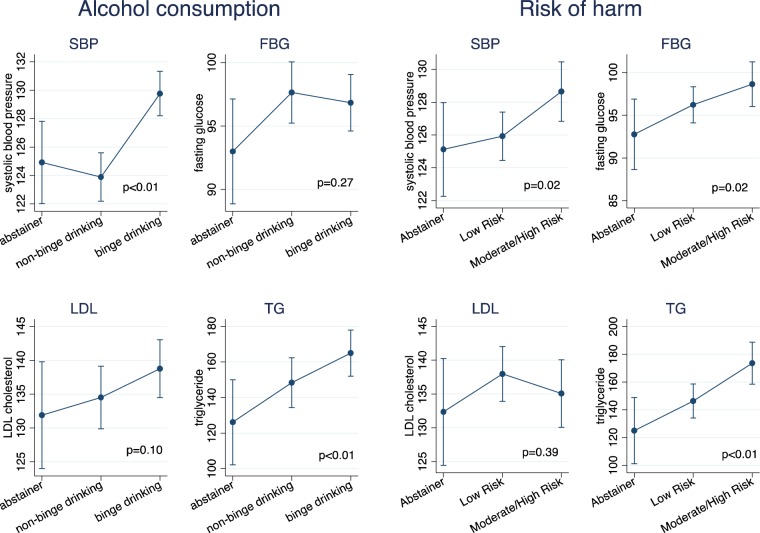
Heavy alcohol use, risk of harm from alcohol use and physiological risk factors for NCDs among men (N = 732). p-value obtained from overall likelihood ratio test between exposure of interests (alcohol consumption or risk of harm) and NCD risk factor using linear or logistic regression. Error bars represent 95% Confidence intervals.

For physiological risk factors among women, higher risk of harm from alcohol use was associated with higher levels of triglycerides and marginally associated with higher levels of systolic blood pressure. Increased risk of harm from alcohol use was not significantly associated with higher levels of blood glucose or LDL cholesterol (Fig. 3). While for men, higher risk of harm from alcohol use was associated with higher levels of systolic blood pressure, blood glucose and TG, but not associated with LDL cholesterol (Fig. 4).

### Heavy alcohol use and risk factors for NCD

In the adjusted model, among women, binge drinking was associated with current smoking and higher levels of BMI but not associated with inadequate fruit/vegetable consumption and inadequate physical activity (Fig. 1). For men, binge drinking was associated with current smoking, inadequate physical activity and higher BMI but not associated with inadequate fruit/vegetable consumption (Fig. 2).

For physiological risk factors among women, binge drinking was associated with higher levels of systolic blood pressure, triglycerides but not associated with blood glucose, LDL cholesterol (Fig. 3). For men, binge drinking was associated with increased blood pressure, increased TG and weakly associated with increased LDL cholesterol but not associated with blood glucose (Fig. 4).

## Discussion

Using ASSIST as a screening tool, higher risk of harm from alcohol use was associated with two of the eight NCD risk factors among women and five of the eight NCD risk factors among men. The same result in both sexes found in systolic blood pressure and TG. Using WHO STEPS as a screening tool, heavy alcohol consumption was associated with four of the eight NCD risk factors among women and six of the eight NCD risk factors for men.

WHO STEPS detected frequency and pattern of alcohol use through measurements of standard drink that reflect volume of alcohol use. Binge drinking has been shown to be associated with other NCD risk factors^14,15^. Previous literature in Thailand also found that drinking four or more glasses per occasion was associated with elevated risk of NCD^6^. ASSIST, however, detected risk of harm from alcohol use by assessing the impact of alcohol consumption on psychosocial aspects such as social problems and concern of peers^10^. The different aspects of alcohol use detected by the two instruments can help explain the difference in associations with other NCD risk factors.

The study found that the association between risk of harm and other NCD risk factors differed by gender. The risk of harm from alcohol use correlated with higher volume of alcohol use among men, which potentially translates to higher levels of NCD risk factors. However, higher risk of harm from alcohol use in women did not correlate with volume and thus did not correlate with as many NCD risk factors as men. There are multiple factors which may contribute to these findings.

Firstly, biology and alcohol metabolism are different between men and women. Women are at higher risk of breast cancer than men^16^. Moreover, women have less total body water than men who have the same body weight. Therefore, given the same amount of alcohol, blood alcohol concentration is higher in women, potentially putting women at high risk from alcohol related diseases such as alcoholic liver diseases^17–19^ For women, small volumes of alcohol may result in intoxication. ASSIST is sensitive to detect risk of harm from alcohol use such as intoxication and its effect. This is supported by the literature that women are at higher risk of alcohol dependence than men, given similar pattern/volume of use^20^. In addition, women eliminate alcohol faster than men^18^, thus literature has shown that the risk of alcohol- related NCD such as stroke, cirrhosis or some cancer are less among women^21–24^. Thus, while there was high risk of harm from alcohol use, its longer-term effects in terms of other NCD were less likely to be observed among women.

The social norm in Thailand may also play a role why women may be at higher risk of harm from alcohol use despite low volume. Thai society tends to stigmatize women consuming alcohol^25,26^. In addition, alcohol use among Thai men is considered normal when socializing^27^. Women, however, are more likely to use alcohol when they have negative moods or emotions^17,28^. All these factors contribute to why women may be at higher risk of harm from alcohol use despite low volume comsumption.

The study is not without limitations. Due to the nature of the cross sectional design, we could not establish causality between alcohol consumption and NCD risk factors. The outcomes of our study rely on self-reported behavioural risk factors for NCD. However, the results also seemed to translate to other physiological markers of NCDs. Quantification of alcohol use may be affected by measurement bias and social desirability bias but the study used third party interviewers who were trained on the questionnaires to quantify volume of alcohol use. This help increased the accuracy of conversion to standard drinks and help minimize such bias. However, recruiting our sample from a single center might limit the generalizability of our results.

Because harmful use of alcohol is a significant contributor to the global burden of NCD^29^, WHO suggested a target of an at least 10% reduction in the harmful use of alcohol within a national context^30^. Since our study found evidence that both volume and risk of harm from alcohol consumption correlated with NCD risk factors, there are some practical implications. Firstly, for men, ASSIST may be an easier screening tool to be incorporated as part of NCD programs as practitioners do not have to worry about the accuracy of the alcohol quantification and conversion to standard drinks. However, among women, using tools to quantify volume may still be needed. Secondly, the study supports the literature that when healthcare providers encounter patients with various NCDs risk factors, alcohol consumption should be assessed^31,32^. When assessing volume of alcohol use, measures of harm related to alcohol should also be assessed as these two measurements of harmful alcohol use did not correlate well among women.

## Data Availability

The datasets generated during and/or analysed during the current study are available from the corresponding author on reasonable request.
